# COVID-19-Induced Fatal Thrombotic Thrombocytopenic Purpura in a Healthy Young Patient

**DOI:** 10.1155/2022/2934171

**Published:** 2022-12-06

**Authors:** Mariana Codevila Buere Pereira, Bruna Ruschel, Bruna Schneider, Vitor Salomão Gonçalves Melo de Melgar, Tatiana Helena Rech

**Affiliations:** ^1^School of Medicine, Universidade Federal do Rio Grande do Sul, Porto Alegre, RS, Brazil; ^2^Intensive Care Unit, Hospital de Clínicas de Porto Alegre, Porto Alegre, RS, Brazil; ^3^Neurology Division, Hospital de Clínicas de Porto Alegre, Porto Alegre, RS, Brazil; ^4^Graduate Program in Medical Sciences: Endocrinology, Universidade Federal do Rio Grande do Sul, Porto Alegre, RS, Brazil

## Abstract

Since the global coronavirus disease 2019 (COVID-19) pandemic began, findings indicate that severe acute respiratory syndrome coronavirus 2 (SARS-CoV-2) might induce autoimmune disorders. Thrombotic thrombocytopenic purpura (TTP) is a devastating disease if not emergently treated. It presents with severe thrombocytopenia, microangiopathic hemolytic anemia, and neurologic findings with or without renal insufficiency. The antibody-mediated reduced activity of the disintegrin and metalloproteinase with a thrombospondin type 1 motif, member 13 (ADAMTS13) induces the accumulation of ultrahigh-molecular-weight multimers of von Willebrand factor, leading to platelet aggregation and thrombosis. TTP can be an unusual presentation of COVID-19 disease mediated by the virus-induced immune response. We report a case of a healthy young patient presenting with the classic TTP pentad a few days after a diagnosis of COVID-19 confirmed by a positive SARS-CoV-2 RT-PCR test. The patient was initially treated with high-dose methylprednisolone and fresh frozen plasma until she was transferred to a tertiary care facility and plasma exchange was available. She evolved with a malignant ischemic vascular accident and was declared brain-dead 24 hours after the first plasma exchange section.

## 1. Background

The coronavirus disease 2019 (COVID-19) first emerged in December 2019 in Wuhan, China, and has caused a significant global increase in hospitalizations and deaths since then. COVID-19 is caused by the severe acute respiratory syndrome coronavirus 2 (SARS-CoV-2) that may lead to a wide spectrum of symptoms, from asymptomatic disease to acute respiratory failure [1]. Moreover, the virus might trigger autoinflammatory, autoimmune, and immunological conditions [2].

Thrombotic thrombocytopenic purpura (TTP) is a disorder of platelet aggregation resulting in microangiopathic hemolytic anemia evidenced by fragmentation of red blood cells, consumption of platelets, and occlusion of the microvasculature caused by a deficiency of the disintegrin and metalloproteinase with a thrombospondin type 1 motif, member 13 (ADAMTS13), a metalloprotease that cleaves von Willebrand (vWF) factor multimers. The vWF is normally secreted as ultralarge multimers, cleaved by ADAMTS13 to smaller multimers [3]. The accumulation of ultralarge multimers favors platelet aggregation and thrombosis [3, 4]. TTP is a condition that classically presents with a pentad of features: fever, purpura or hemorrhage associated with thrombocytopenia, hemolytic anemia with schizocytes on the peripheral blood smear, neurological manifestations, and renal dysfunction [5]. Microvascular occlusion results in organ damage, most notably of the kidney and brain [4]. The combination of microangiopathic hemolytic anemia and thrombocytopenia without other explanation is sufficient to diagnose and treat TTP as a hematological emergency, due to its fatal feature if untreated [4, 6].

TTP can be inherited or secondary to antibody formation (idiopathic, medication-related, or infection-triggered) [7]. Studies suggest that the development of coagulopathy in the course of COVID-19 is a result of the inflammatory response to SARS-CoV-2. Besides, COVID-19 is clearly associated with thrombotic events [8, 9].

Despite limited understanding, COVID-19 can have immune system hyperresponsiveness. Several studies have already shown that COVID-19 can be associated with autoinflammatory and autoimmune diseases like Kawasaki disease, Guillain-Barré syndrome, autoimmune hemolytic anemia (AIHA), and immune thrombocytopenic purpura (ITP) [2].

Therefore, COVID-19 has a link with TTP, a rare disease that presents with a deficiency of ADAMTS13. A reduced ADAMTS-13/vWF ratio has been described in patients with COVID-19, generating a prothrombotic milieu that is associated with disease severity and prognosis, including reduced survival [10].

## 2. Case Report

A 23-year-old white female with no previous medical history had a positive SARS-CoV-2 nasal swab RT-PCR test with few symptoms at the end of July 2021. On August 11, she was evaluated in the emergency department because of hemiparesis associated with dysarthria lasting for ten minutes. The initial laboratory screening revealed severe thrombocytopenia, hemolytic anemia, and hematuria. She was admitted to a community hospital for investigation, and the computed tomography (CT) angiography of the brain was normal.

Five days after admission, she evolved with a decreased mental state and was intubated for airway protection. The magnetic resonance imaging of the brain revealed hyperacute ischemic stroke with small spots of diffusion restriction in the right internal capsule and in the anterior cortex of the left precentral gyrus. At this time, a SARS-CoV-2 antigen test came out negative.

**Figure 1:**
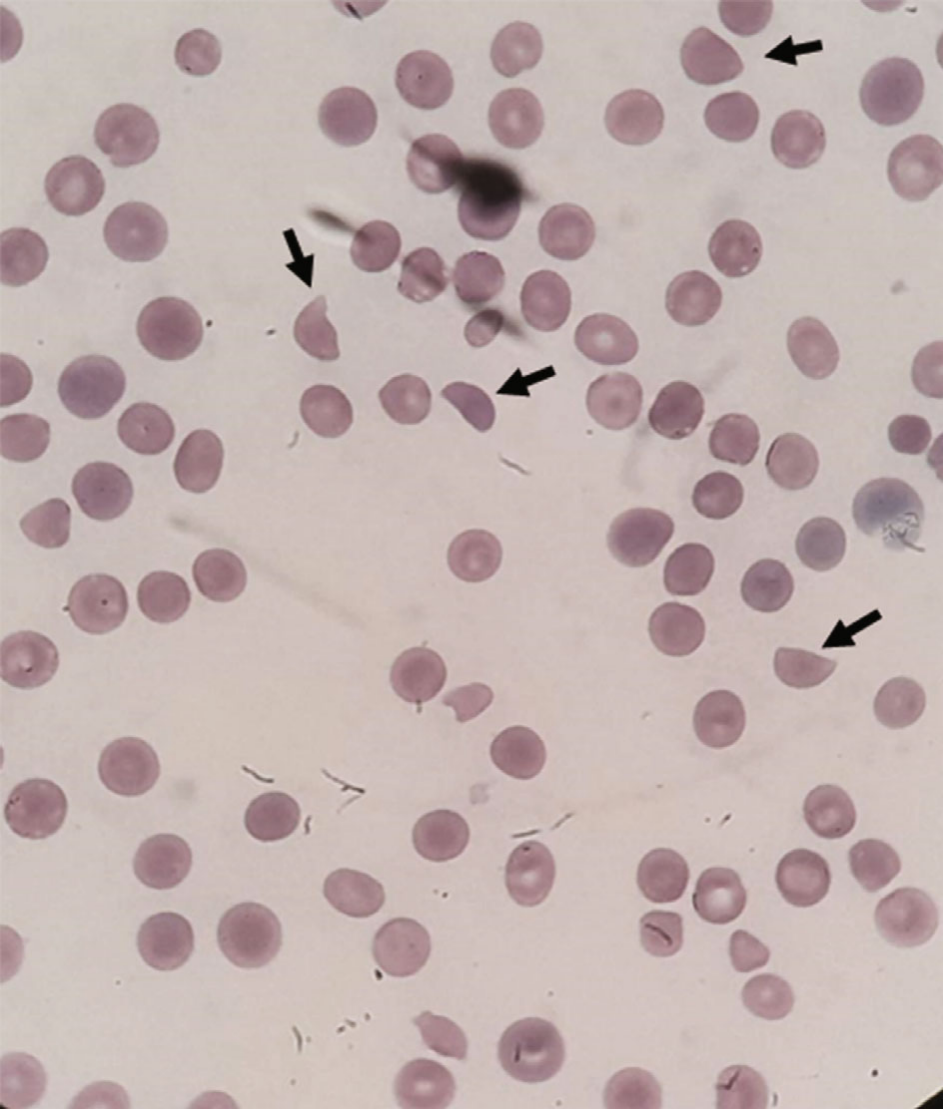
Peripheral blood smear examination revealing numerous schistocytes (arrows). These findings are consistent with microangiopathic hemolytic anemia, one of the features of the classic TTP pentad.

TTP was suspected, and she received methylprednisolone at a dose of 125 mg intravenous every 6 hours and daily fresh frozen plasma to replace ADAMTS13, since plasma exchange was not available at the hospital facility. She was then transferred to our university hospital for plasma exchange on August 19. Her physical examination revealed a patient in a coma with symmetrical miotic pupils, pallor of mucous membranes, and jaundice. Her vital signs were stable, with a blood pressure of 124/76 mmHg and a heart rate of 110 beats per minute and with no arrhythmias and normal heart sounds. She was sedated on invasive mechanical ventilation (MV) with low-oxygen-inspired fraction and clear lung fields. Her abdominal examination was normal, but hematuria was present. No skin lesions were detected. Blood glucose was 12.43 mmol/L and the body temperature was at 38.8°C. The complete blood count revealed anysocytosis, polychromatophilia, and fragmented red blood cells (Figure 1). Creatinine was 106.1 umol/L, D-dimer was 7.8 mg/L, and prothrombin time (PT) and activated partial thromboplastin time (aPTT) were normal range (Table 1). HIV and pregnancy tests were negative. A new SARS-CoV-2 nasal swab PCR test was ordered.

Facing the TTP classical pentad, we applied the PLASMIC-score, a validated tool to stratify patients with thrombotic microangiopathy according to their risk of having severe ADAMTS13 deficiency [11]. She scored 6 points (scale of 0 to 7), considered a high risk. Then, plasma exchange was immediately started.

**Figure 2:**
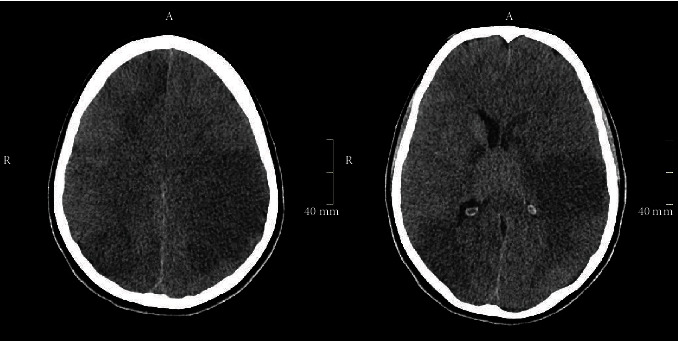
Computed tomography of the brain evidencing a large hypodense area with effacement of the cortical sulci and midline shift, suggesting a malignant ischemic stroke.

The patient became severe tachycardic with high fever, sudden anisocoria was noted, and sedation was stopped. The CT of the brain evidenced a large hypodense area with effacement of cortical sulci and midline shift, tortuosity of the orbital optic nerve, and blurring of the peduncle, midbrain, and cisterns (Figure 2), signs compatible with intracranial hypertension. Taken together, the findings suggested ischemic events evolving in multiple vascular territories.

Few hours later her physical exam revealed bilaterally fixed mydriasis, absence of brainstem reflexes, and hemodynamic instability. Brain death was confirmed and the patient was declared dead less than 24 hours after admission. The state Organ Procurement Organization was notified, but the SARS-CoV-2 rapid PCR test came out positive, precluding organ donation.

## 3. Discussion

We described here a case of a young healthy patient presenting with the classical TTP pentad few days after a diagnosis of an almost asymptomatic COVID-19, confirmed by PCR test. Recently, an increasing number of cases of autoimmunity possibly COVID-19-induced have been reported in the literature, suggesting a link between COVID-19 and an altered immune function [10].

Other hematologic autoimmune diseases besides TTP have been described in patients infected with SARS-CoV-2, including AIHA, ITP, antiphospholipid syndrome, autoimmune neutropenia, and Evans syndrome [2, 12]. A systematic review with 94 patients who developed hematologic autoimmune disorders during the course of COVID-19 infection revealed the development of ITP in 58% of patients, AIHA in 23%, and TTP in 3%. However, as opposed to our healthy young patient, they were elderly with several comorbidities. As far as we know, we present here the first case of a healthy young patient with TTP and a fatal outcome after COVID-19. Our patient presented with hematologic autoimmune symptoms two weeks after COVID-19 infection, around the same time point previously reported in the literature (11.8 ± 7.1 days**)** [12].

Mild to severe degree of thrombocytopenia has been demonstrated in one-third of patients with SARS-CoV-2 infection, and COVID-19 should be considered in the setting of an acute reduction in platelet count with no other obvious cause [13]. Besides, thrombocytopenia is closely related to prognosis in patients with COVID-19. At hospital admission, patients with thrombocytopenia had a three times higher mortality than patients without thrombocytopenia [14]. A retrospective study with 1,476 patients with COVID-19 reported the presence of thrombocytopenia in 72.7% of patients who died compared to 10.7% in those who survived (*p* < 0.001) [15]. The pathogenesis of SARS-CoV-2 immune thrombosis has not yet been completely understood, but ADAMTS13 inhibitors induced by COVID-19 seem to contribute to thrombotic microangiopathies [16]. There is evidence that endothelial activation increases production of vWf in COVID-19 patients, which may exceed the ADAMTS13 processing. The relative deficiency of ADAMTS13 leads to the formation of a large TTP-like vWf multimer with potential to explain the immunothrombosis [17]. Moreover, ADAMTS13/vWf antigen ratio predicts disease severity and mortality in COVID-19 disease [17].

Patients infected with SARS-CoV-2 associated with specific genetic signatures (still unrevealed) may evolve with more vigorous immune responses, presenting with immune system hyperactivation and a dramatic increase in immune-inflammatory parameters, which can lead to autoimmunity and cytokine storm [18]. This suggests that COVID-19 infection might explain some immune and inflammatory manifestations with autoimmune features, leading to poor outcomes and high mortality. We believe this was the case resulting in the death of our healthy young patient: a strong deleterious combination of a genetic autoimmune signature with an environmental viral trigger.

## 4. Conclusions

Since the beginning of the COVID-19 pandemic, several hematologic autoimmune disorders associated with SARS-CoV-2 were described, including cases of TTP. Our patient presented a sequence of events that suggests that COVID-19 played a direct role in triggering autoimmunity. Despite being a rare autoimmune disease in COVID-19 patients, TTP should be considered in the differential diagnosis of thrombotic microangiopathy in the presence of SARS-CoV-2 infection. Timely monitoring of platelet decline is especially important because autoimmune manifestations present about one to two weeks after the first signs of the SARS-CoV-2 infection, and the platelet count is an important indicator of poor prognosis. This case report reinforces the importance of considering an immune etiology in the setting of acute and profound platelet decrease in COVID-19 patients despite of the absence of comorbidities, as it may lead to unfavorable outcomes, including death.

## Figures and Tables

**Table 1:** Sequential laboratory tests.

Biochemical parameters	Reference range	August 11^th^	August 16^th^	August 19^th^
Hemoglobin (g/dL)	12-16	7.5	6.4	6.5
Hematocrit (%)	37-47	21	18	19
Reticulocytes (%)	0.5-1.5	16	—	16
Mean corpuscular volume (fL)	80-98	91	92	102
Platelet count (10^3^/*μ*/L)	150-450	9	6	6
Leukocyte count (10^3^/*μ*/L)	4.5-11	8.7	11.5	18.6
aPTT (seconds)	25-35	19	17	26
PT (seconds)	11-13	11	11	15
INR (IU)	<1.2	1.0	1.0	1.2
Fibrinogen (g/L)	2-4	2.01	—	3.1
AST (U/L)	10-40	38	—	484
ALT (U/L)	10-40	16	—	931
Bilirrubin, total (umol/L)	5.1-17.1	34.2	—	114.57
Bilirrubin, direct (umol/L)	1.7-5.1	13.68	—	71.82
Bilirrubin, indirect (umol/L)	3.4-12	20.52	—	42.75
Urea (mmol/L)	2.9-7.1	7.5	27.14	52.49
Creatinine (umol/L)	44-97	61.89	88.42	106.1
LDH (U/L)	80-225	849	2356	3711
SARS-CoV-2 test result	Negative	—	Negative (antigen)	Positive (nasal swab PCR)

ALT: alanine aminotransferase; aPTT: activated partial thromboplastin time; AST: aspartate aminotransferase; INR: international normalized ratio; LDH: lactate dehydrogenase; PT: prothrombin time.

## Data Availability

The reference data used to support the findings of this study are included within the article.
